# Molecular Analysis by Microsatellite Markers of Goji Plants (*Lycium barbarum* L.) Grown in Central Italy Reveal Genetic Distinction from Both *L. barbarum* and *L. chinense* Species

**DOI:** 10.3390/plants14081182

**Published:** 2025-04-10

**Authors:** Letizia Poggioni, Claudio Cantini, Giorgio Binelli, Giampiero Cai, Veronica Conti, Lavinia Mareri, Marco Romi, Chiara Piccini

**Affiliations:** 1 Department of Life Sciences, University of Siena, 53100 Siena, Italy; letizia.poggioni@icloud.com (L.P.); giampiero.cai@unisi.it (G.C.); veronica.conti@unisi.it (V.C.); laviniamareri@hotmail.it (L.M.); marco.romi@unisi.it (M.R.); 2 National Research Council of Italy, Institute for BioEconomy (CNR-IBE), 58022 Follonica, Italy; claudio.cantini@ibe.cnr.it; 3 Department of Biotechnology and Life Sciences, University of Insubria, 21100 Varese, Italy; giorgio.binelli@uninsubria.it

**Keywords:** DNA analysis, SSR markers, genetic structure, cultivar, clonal selection

## Abstract

*Lycium barbarum* L. is a shrub native to China. It produces berries that are high in nutraceutical value. Its commercial appeal has led to the development of new plantations in Italy over recent decades. The majority of cultivated goji plants are derived from local nursery seedlings without the selection of varieties or clones. This study used nine simple sequence repeats (SSRs) from *Lycium chinense* L. to analyze the genetic structure and variability of heterogeneous, seed-derived accessions cultivated in an orchard located in central Italy (from here on referred to as field). The results were compared to accessions of known origin (LB, *Lycium barbarum*; LC, *Lycium chinense*). The study aimed to determine the genetic origin of seedlings and assess the feasibility of using microsatellite markers for individual fingerprinting. It also aimed to propagate the most adapted, productive plants while ensuring traceability and protection of potential clones throughout the production chain. The SSR markers used revealed that the field accessions were genetically distinct from both the *L. barbarum* and *L. chinense* accessions, whose seeds came from different European Botanical Gardens. The mean observed heterozygosity (H_o_) across the three groups was 0.356, higher than the mean expected heterozygosity (H_e_) of 0.314. The values of the inbreeding coefficient (F_IS_) ranged from −0.25 (field) to 0.05 (LC), confirming the high genetic variability in our dataset. The fixation index (F_ST_) was 0.234, indicating medium to high genetic differentiation. The Bayesian analysis revealed three distinct clusters, indicating that three gene pools influenced the genetic structure of the studied populations. The orchard accessions form a distinct population, most likely a *L. barbarum* landrace, descended from two distinct ancestral populations that differ from the two known species. Our findings preliminarily lay the groundwork for the protection of some clonal lines of goji accessions for use in future planting more suited to the Mediterranean climate. This study also serves as a foundation for a more thorough characterization of cultivated *L. barbarum,* allowing for traceability and sustainable management of the genetic resource.

## 1. Introduction

The comprehensive analysis of DNA sequence polymorphism is essential for genetic research. Within this framework, molecular markers are critical tools for accurately assessing genetic variability. These markers have made significant advances in the genetic characterization of crop species, allowing for improved crop breeding programs, increased resistance to pathogens, and agronomic traits optimization. Researchers can use molecular markers to dissect the genetic architecture of plants, helping to develop innovative strategies for sustainable agriculture and food security [1]. In several crops, molecular markers such as restriction fragment length polymorphisms (RFLPs), random amplification polymorphic DNA (RAPD), amplified fragment length polymorphisms (AFLPs), and microsatellites or simple sequence repeats (SSRs), have been developed [2,3,4]. Microsatellites have proved to be very useful for a wide range of applications in plant genetics due to their reproducibility, multiallelic nature, codominant inheritance, relative abundance, and good genome coverage [5].

Indeed, SSR markers have become an indispensable tool for exploring genetic diversity, as demonstrated in studies on rice [6], pepper [7], and torch ginger [8]. Researchers have used these markers to assess genetic variability, establish germplasm fingerprints, analyze dominance patterns, trace germplasm migration, evaluate gene flow, construct genetic linkage maps, and perform phylogenetic analyses [9]. Given its abundant wild resources and complex genetic makeup [10,11], *Lycium* is a particularly compelling subject for genetic investigation.

Information on genetic variation is thus fundamental for the evaluation of genetic resources, as it is a source of knowledge about the molecular structure of plants and can be used as a basis for plant selection as well as the effective evaluation, conservation and use of germplasm of any population [12].

Genetic investigation approaches have also been developed for *Lycium* (fam. *Solanaceae*) species to differentiate the species objective of this work, *Lycium barbarum* (commonly known as goji), from other related species, since they are difficult to distinguish using morphological, histological, and biochemical approaches [13]. *L. barbarum* and the related species *Lycium chinense*, both endemic to Korea, Japan, and China, have been widely used in Eastern traditional medicine for more than 2000 years because of their emmenagogue, diuretic, antipyretic, tonic, aphrodisiac, hypnotic, and hepatoprotective properties, and today there is a growing demand in the health food market [14,15].

The earliest study on this topic used the Random Amplified Polymorphic DNA (RAPD) technique to distinguish *L. barbarum* from other closely related species of the same genus [16]. Sequence Characterized Amplified Region (SCAR)-based approaches have been subsequently developed for *Lycium* species to differentiate *L. barbarum* from *L. chinense var. potaninii* samples, detecting two characteristic bands on RAPD profiles, one of which was amplified only in *L. barbarum* samples [16,17].

Kwon et al. [18] isolated and characterized 21 polymorphic microsatellite markers developed in the *L. chinense* Mill. These markers generated 86 alleles in 30 of the analyzed accessions. All loci were successfully amplified for all *L. barbarum* accessions. This work continues to serve as the foundation for microsatellite markers, and a subset of these was used in this study, though not all published markers performed as well as reported. Chung et al. [19] attempted to discriminate among the different cultivars of *L. barbarum* using the SSR markers developed by the aforementioned work [18]: 41 cultivars from Korea and China were analyzed using 10 markers, revealing a total of 61 alleles. The combination of these five microsatellites distinguished all identified cultivars, but only half of the markers were effective for discrimination.

With the rapid development of modern molecular methods, DNA fingerprinting techniques are supplementing traditional anatomical and physiochemical methods for the identification of species relationships. The genetic diversity and population structure of *L. chinense* accessions was evaluated using the polymorphic SSR markers [18] detecting a total of 108 alleles [20]. More recently, double digest restriction-associated DNA sequencing (ddRAD) was successfully applied for building a densely saturated genetic map of *L. barbarum* [21], which was also used for the localization of quantitative trait loci (QTLs) controlling photosynthetic traits. Also, a study by Gao et al. [22] used 16 highly polymorphic SSR primer pairs to assess the genetic diversity of *Lycium* germplasm from northwest China, observing a wide genetic variation within the analyzed populations, indicating considerable internal diversity.

Regarding the transcriptome characterization, in 2017, Chen et al. [23] carried out an in-depth study on the species *L. barbarum*, aimed at providing a resource for functional gene extraction and developing EST-SSR markers that can be used for genetic diversity assessment, linkage map construction, the fine-mapping of crucial genes, and marker-assisted breeding.

Goji berries have recently emerged as a popular commercial crop in Italy. In 2016, for example, our country produced approximately 50 tons of fruit, making it the largest producer in Europe. The regions where the majority of most Italian goji is produced are Veneto in northern Italy; Tuscany, Umbria, and Lazio in central Italy; and Puglia and Calabria in southern Italy [24]. Many farms established new plantations to produce a fully traceable product in accordance with national legal requirements. However, a significant challenge persists because many plants in actual orchards are grown from seeds, with little or no knowledge of their origin or genetic characteristics.

This lack of genetic information, combined with extreme variability among individuals, can degrade berry quality, potentially reducing yield and nutritional value. Furthermore, the lack of distinct genetic lines maintained through self-propagation complicates understanding of productive capability, adaptability, and resilience to various environments. To address this issue, investment in genetic research and development is required to ensure the cultivation of high-quality, resilient plant lines that meet both consumer and regulatory expectations.

The purpose of this study was to use a set of nine polymorphic microsatellite loci from Kwon et al. [18] for the molecular characterization of *L. barbarum* accessions grown in an orchard located in central Italy. The first objective of this research was to better understand the genetic origin and structure of the population under study by comparing orchard accessions to those of known origin.

On a practical level, the final goal of this work was to determine whether the set of markers could be used for the genetic characterization of all individuals in the orchard. The precise characterization of the accessions using molecular markers was critical because our group is interested in studying the performance of the most productive/adapted accessions in the orchard. The final goal of the analysis was to characterize an accession to be used to start a line of clones selected because they are more suitable for cultivation in Mediterranean conditions. This research was also motivated by the possibility of protecting the selected genetic lines along the entire production chain, from nursery to fruit and secondary products.

## 2. Results

The number of expected alleles (A_e_) ranged from a minimum of 2 to a maximum of 6, while the number of observed alleles was from 2 to 5. The number of alleles observed in the field population ranged from a minimum of 1 to a maximum of 4, while the LB population was from a minimum of 1 to a maximum of 3 and the LC from 1 to 2 (Table 1).

The GB-LCM-167 locus showed the highest number of alleles (n = 5), while GB-LCM-075 and GB-LCM-119 were the ones with the lowest number (n = 2). Only GB-LCM-25, GB-LCM-104, and GB-LCM-166 showed the same number of alleles compared to the expected number (n = 3) and GB-LCM-199 showed instead a higher number than expected (n = 3). The GB-LCM075 locus was found to be the only one always homozygous, with a peak at 187 bp for the LB and LC population and at 230 bp for the field population. The GB-LCM-021 locus showed the widest range (78 bp and 4 alleles), while the GB-LCM-119 locus showed the narrowest range (4 bp and 2 alleles).

Table 2 shows the results of genetic variability and differentiation analysis. The three examined groups showed higher observed heterozygosity (H_o_) values than the expected (H_e_) based on Hardy–Weinberg equilibrium (HW): in fact, the values of H_o_ ranged from 0.259 to 0.443 (mean value = 0.356) versus values of H_e_ that ranged from 0.272 to 0.355 (mean value = 0.314).

The three groups showed a similar mean number of alleles per locus (N_A_): the LB population obtained a mean number of 2.00, the LC obtained 1.67, and the field obtained 3.00, and overall, the mean number of alleles per locus was 2.22.

The values of the inbreeding coefficient F_IS_, which indicates the variance of allele frequencies within the population, showed values from −0.25 (field) to 0.05 (LC): the negative value shows an excess of heterozygous accessions in the populations, confirming the high genetic variability present in our dataset.

The overall F_ST_ is 0.234 (confidence interval at 0.95 level: 0.046–0.391) indicating moderate to high genetic differentiation, mostly due to the Italian accessions as shown by the pairwise F_ST_ analysis (Table 3). The LB and LC populations are not differentiated at all, while the field accessions present high and significant F_ST_ values versus both LB and LC accessions.

**Figure 1:**
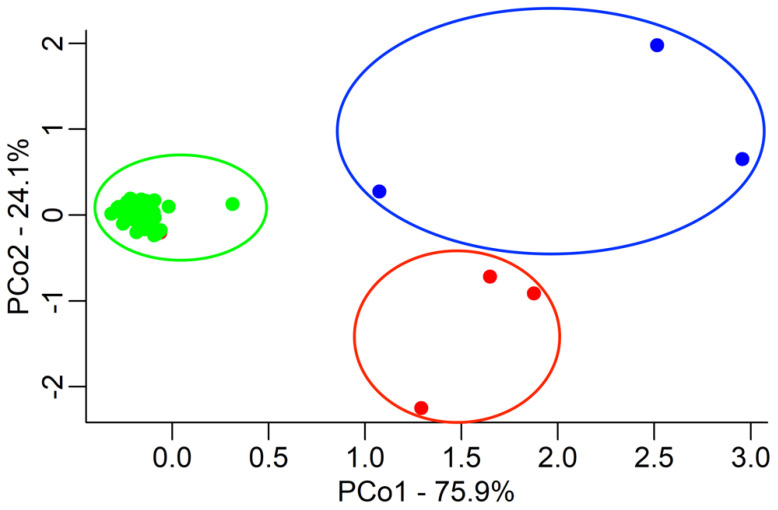
Principal Coordinate Analyses (PCoAs) were performed on the results of the three populations: LB (red), LC (blue), and field (green). Each accession is represented by a point in a two-dimensional space represented by the first two PCos, which, respectively, explain 75.9% (PCo1) and 24.1% (PCo2) of the total variations. The Euclidean distance between accessions is based on their genotypic differences.

The Principal Coordinates Analysis (PCoA) (Figure 1) highlighted that the LB accessions cluster together (*L. barbarum* species) and two of them are perfectly identical, except for one element, which showed characteristics more similar to the field population. The three LC accessions formed a second cluster, whilst the field population (green), which was supposed to be composed exclusively of *L. barbarum*, formed clusters apart from both LB and LC.

In this regard, the population structure in the three identified groups (Table 4) was studied using the Bayesian approach implemented by STRUCTURE. First, the most probable number of source clusters (K, or homogeneous gene pools) based on ΔK (i.e., the second-order change rate of the likelihood function with respect to K) was estimated to be equal to three, thus indicating that three gene pools shaped the genetic structure of the analyzed populations. Based on this, the final proportion of each of the three hypothetical gene pools present in each population was obtained.

**Figure 2:**
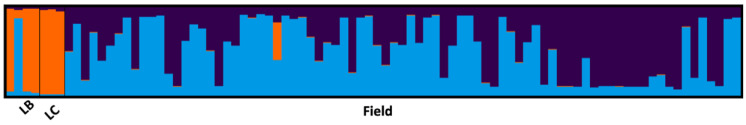
Bayesian analysis of the genetic structure of the populations on the single accession genome. Each bar represents a single accession genome. The different colors represent the percentage of the genome from each of the three detected clusters (light blue, purple, and orange).

Figure 2 shows the bar graph of the Bayesian analysis of population genetic structure based on the genome of each individual accession. Each bar represents the genome of an accession, and the different colors represent the cluster to which it belongs. Almost the all the genomes of the accessions belonging to the LB group and the LC group are derived from a single ancestral population (orange cluster, with the exception of the single accession LBL2, an accession of *L. barbarum* from the Mainz Botanical Garden (Germany)), while the field group presents mixed origins from two other different genetic pools, with just one accession (M8-6, origin unknown, like all the other accessions in the field group) showing the orange component of the other two groups (LB and LC).

## 3. Discussion

Molecular analysis revealed that accessions from the Magliano orchard (field) were genetically distinct from both *L. barbarum* (LB) and *L. chinense* (LC) accessions used for comparison in this study. While LB and LC accessions showed little genetic variation, suggesting a common ancestor, accessions from the field group had a unique genetic composition derived from two distinct ancestral populations. The molecular marker analysis clearly shows that the studied population is a distinct genetic entity, different from both *L. barbarum* and *L. chinense*, and thus, it could potentially represent a valuable reservoir of genetic variability for future breeding and/or selection programs. This finding strongly suggests that the Magliano orchard accessions are most likely one or more local ecotypes that have adapted to their specific environmental conditions.

As a result, the findings are relevant. First, it can be confirmed that the markers used do not effectively discriminate between the related species *L. barbarum* and *L. chinense*, as shown in many studies [18,20,25]. Indeed, from both a morphological and phylogenetic perspective, the two species are found to be highly similar and, as a result, they are frequently used interchangeably in the herbal and ethnic markets because of their comparable nutraceutical properties [26]. For example, the fruits of *L. barbarum* are significantly sweeter and more palatable than those of *L. chinense*, which is occasionally used as an adulterant [14]. The current set of SSRs is unlikely to distinguish between the two species, because microsatellites focus on neutral variability [27] rather than the genomic regions that control quantitative traits like sweetness and overall taste. The most recent genetic map of goji, based on ddRAD sequencing, was used to map QTLs controlling several photosynthesis-related traits [21], but not other phenotypic traits with immediate commercial value. The use of a large number of SNPs, combined with the use of classical barcoding information such as the ITS2 region, will most likely represent a breakthrough in our ability to discriminate between *Lycium* species and ecotypes, as supported by Xin et al. [25].

A value of F_ST_ close to 0.25 is considered indicative of a high degree of genetic differentiation among populations [28]: the overall F_ST_ was 0.234 (with a confidence interval at 0.95 level of 0.046–0.391) mostly due to the Italian accessions. The LB and LC populations are not differentiated at all, while the field accessions present high and significant pairwise F_ST_ values in comparison to both LB (0.159) and LC (0.357). These results are in line with the study by Gao et al. (2023) [22] on the Chinese wild populations of *L. barbarum*, where the calculated F_ST_ value was 0.27 among the studied populations. The LB and LC accessions share Asian ancestry [29], as evidenced by their lack of differentiation and the Bayesian analysis revealing the same gene pool of origin. Furthermore, the fact that the SSR markers used, which were originally developed in *L. chinense*, work well in *L. barbarum* [18] and in our Italian field group accessions further supports this.

It is worth noting that an accession from the Mainz Botanical Garden (sample LBL2) shared a similar common ancestry with several field accessions, implying a possible shared origin as a cultivar or landrace. However, more research is needed to confirm this hypothesis due to the ambiguous origins of all goji accessions grown in Europe, a factor suggested, albeit indirectly, by the current data. The multifaceted origin of the cultivated goji can also be inferred by the work by Zhang et al. [30], which studied nineteen accessions, including four wild species (*L. ruthenicum* Murr., *L. barbarum* L., *L. chinense* Mill. var. *potaninii* Pojark., and *L. yunnanense* Kuang), one mutant with wild white fruits, and a number of native species, landraces, crosses, and mutants attributed to the species *L. barbarum*. The four species were found to be genetically similar, and the various landraces shared a common ancestor in *L. barbarum*, which could explain why they are so closely related. The low diversity of native species could be attributed to the long-term artificial directional selection, which has resulted in the elimination of lines (and, consequently, genes) deemed unsuitable for agricultural purposes, resulting in a convergence in similar morphology (“convergent evolution”) among native species with divergent ancestors. This study proposes that, in order to ensure the long-term advancement of Chinese goji, additional goji germplasm material be incorporated into diverse breeding programs to enhance the gene pool and prevent deterioration in quality caused by inbreeding.

A comparable scenario is observed in *L. chinense*, as demonstrated by Zhao et al. [20]. Utilizing 18 SSR markers derived from the aforementioned research by Kwon et al. [18], the study assessed the genetic diversity and population structure of 139 *L. chinense* accessions. The analysis revealed that the accessions exhibited at least 70% ancestry in common with one of the three inferred groups. In addition to the groups identified in this analysis, 8.2% of the accessions showed evidence of descent from a mixed population, which is likely due to recent breeding, domestication history, and resource exchange, all of which have had a significant impact on the genetic structure of the various accessions. For example, human-mediated gene flow can occur within a population as a result of breeding, resulting in a significant amount of variation attributed to within-group differences (84.7%) rather than among the three groups suggested by Zhao et al. [20].

It is thus possible that in the future genetic information will be used to characterize the accessions cultivated in Europe, allowing for the more precise indications of their origin and the species and landrace to which they belong, together with a list of morphological descriptors of the plants and the nutraceutical profile of the fruits and leaves. Further research could be carried out to establish breeding and conservation programs, as well as supply chains, to ensure the traceability and long-term sustainable use of resources derived from these accessions.

Our group has already published evidence based on the orchard accessions in Poggioni et al. [31]. However, some of the accessions analyzed in this study have superior agronomic characteristics to the others and are worthy of attention. Our intention is to use the best-performing and most environmentally adapted individuals as mother accessions to begin propagation and the production of cloned lines that will be used for further research in orchards more suitable for goji production in the Mediterranean climate.

## 4. Materials and Methods

### 4.1. Plant Materials

Eighty-one *L. barbarum* accessions were selected within an Italian orchard cultivated in central Italy at Magliano in Toscana (42°38′29.75″ N, 11°16′17.45″ E) by the Bragaglia Farm. The 1 ha orchard was planted in 2010 at a spacing of 1.6 m × 2.8 m, and the plants were managed as a hedgerow and were pruned every year. The orchard is cultivated in rainfed conditions, in accordance with organic EU farming directives. The climate of the area is typical Mediterranean with data available from a public thermo-pluviometric station (Poggio Perotto, TOS03003011) of the Tuscan Region (SIR network, https://www.sir.toscana.it, accessed on 9 April 2025) located 5 km from the farm. The accessions were purchased from a nursery (Vivaio Vita Verde, Galliera, Bologna, Italy) which obtained the plants from seed. Four accessions of *L. barbarum* and three of *L. chinense* were obtained from CNR-IBE, starting from seeds received from different European Botanical Gardens (Hungary, Slovenia, France, Italy, Latvia, and Germany). The complete list of accessions used in this study is shown in Table S1 in the Supplementary Materials.

Leaf material was sampled during the 2022–2023 season for DNA extraction. To maximize the yield, the new, more tender leaves located in the apical part of the new shoot were hand harvested from each accession. Material was stored at 4 °C and processed within 48 h.

### 4.2. DNA Extraction

DNA for molecular investigations was extracted from leaf material using the cetyltrimethylammonium bromide (CTAB) method of Doyle and Doyle [32], with modifications. A total of 150 mg of young leaves were finely grinded in CTAB buffer (2% CTAB, 200 mM Tris/HCl pH 8.0, 20 mM EDTA, 1.4 M NaCl, 1% PVP K30) and then placed in an incubator at 65 °C for 30 min. After centrifuging, the supernatant was treated on ice with a solution of chloroform/isoamyl alcohol (24:1) and the supernatant was then collected in a new test tube and incubated at 37 °C with 4 uL of RNase A for 15 min. Then, isopropanol was added to the solution to help precipitate the DNA pellet. To improve the quality of the extracted DNA, washing was performed with two types of wash solution: the first composed of 76% ethanol and 200 mM sodium acetate, and the second composed of 76% ethanol and 10 mM ammonium acetate. The DNA pellet was then air-dried for about 10 min and resuspended in 80 μL of ultrapure water (UltraPure^TM^ DEPC-Treated Water, Invitrogen^TM^, Thermo Fisher Scientific Inc., Waltham, MA, USA). The quality and quantity of the resuspended DNA was evaluated by spectrophotometric reading with a Biophotometer^®^ from Eppendorf (Hamburg, Germany) and subsequently by an electrophoretic run on 1.2% agarose gel.

### 4.3. Molecular Markers

The microsatellite markers used were chosen and designed based on the work of Kwon et al. [18]. In a preliminary investigation, some markers were discarded since they were not polymorphic and a final set of nine markers were selected for analyzing both the genetic structure of the population and the variability among samples (Table 5).

### 4.4. PCR and Sequencing Protocols

DNA samples were diluted with ultrapure water (UltraPure^TM^ DEPC-Treated Water, Invitrogen^TM^, Thermo Fisher Scientific Inc., Waltham, MA, USA) to a final concentration of 25 ng/mL. The method used for the amplification of the selected markers is that of Schuelke [33]. For each marker, a mix was created containing H2O MilliQ® (IX 7003/05/10/15 Water Purification System, Merck KGaA, Darmstadt, Germany), Buffer 1X, dNTPs (0.2 mM), Primer Fw (2 pmol), Primer Rv (8 pmol), Primer M13(−21) (8 pmol) (FAM or HEX), 1U Taq, and 2 μL of sample (50 ng of DNA), for a total of 25 μL per well.

PCR amplification conditions were as follows: 94 °C (5 min), then 30 cycles at 94 °C (30 s)/58 °C (45 s)/72 °C (45 s), followed by 8 cycles at 94 °C (30 s)/55 °C (45 s)/72 °C (45 s), and a final extension at 72 °C for 10 min. The verification of the amplification products was performed each time with an electrophoretic run on 1.2% agarose gel. The plates were stored at −20 °C until genotyping.

The analysis of PCR fragments was performed by automatic capillary electrophoresis in fluorescence, on a single capillary sequencer ABI PRISM 310 Genetic Analyzer (Applied Biosystems, Waltham, MA, USA). A 47 cm capillary and POP4 polymer were used for the run. The running module used was GS STR POP4 (1 mL) G5. For the run, 1 mL of PCR in 14 mL of deionized formamide and the GeneScan500LIZ Size Standard were used as an internal molecular weight standard. The analysis was performed using Gene Scan Analysis software v. 5.1 (Applied Biosystems).

### 4.5. Data Analysis

The data obtained from sequencing were processed with the GeneMarker^®^ software (©2020 v3.0.1, SoftGenetics, LLC. All rights reserved). Allele frequencies and observed and expected heterozygosity were estimated as the means of all loci. Weir and Cockerham’s F-statistic estimators [34] were applied to analyze genetic diversity both within and between populations. The deviation of F_IS_ from zero was tested for all loci in all populations using the null hypothesis of Hardy–Weinberg equilibrium via a permutation test based on 1000 replicates. F_ST_ values were estimated to assess the extent of differentiation between populations. Nei’s [35] genetic distance was calculated for pairwise comparisons of populations under an infinite allele model. Principal coordinate analysis (PCoA) was performed based on the pairwise genetic distances between individuals. For these analyses, we used Genetix 4.05.2 [36]. To investigate the presence of genetic structuring in the studied populations, the Bayesian clustering analysis implemented in the STRUCTURE 2.3.1 program was used [37]. First, we determined the most likely number of ancestral gene pools (K) based on nuclear microsatellite genotypes. This was carried out via the K method developed by Evanno et al. [38], using HARVESTER [39].

Additionally, the microsatellites size data were used to calculate the distance among accession by GenAlEx 6.5. program [40] and a phylogenetic tree (see Figure S1 in the Supplementary Materials) was constructed using the Neighbor-Joining (NJ) method via MEGA7 [41]. The tree procedure was solely for the purpose of facilitating the visualization of the results obtained from the fingerprinting and to establish if each accession could be easily identified in comparison to the others.

## Figures and Tables

**Table 1:** List of the SSR markers used for the discrimination of the accessions and their allelic information.

Marker	A_e_	A_o_	A_o_ LB	A_o_ LC	A_o_ Field	Observed Size (bp)	Range (bp)
GB-LCM-021	3	4	3	1	4	240; 248; 264; 318	78
GB-LCM-025	3	3	2	2	2	256; 265; 277	21
GB-LCM-075	5	2	1	1	1	187; 230	43
GB-LCM-087	5	4	3	2	3	211; 217; 222; 225	14
GB-LCM-104	3	3	2	2	3	330; 335; 342	12
GB-LCM-119	3	2	2	1	2	277; 281	4
GB-LCM-166	3	3	2	2	4	207; 211; 217	10
GB-LCM-167	6	5	2	2	3	187; 195; 201; 207; 211	24
GB-LCM-199	2	3	1	2	3	292; 302; 314	22

A_e_ = expected allele number; A_o_ = observed allele number; bp = base pairs.

**Table 2:** Results of genetic variability and differentiation analysis examined through genetic diversity indices (H_e_, H_o_, and F_IS_) for each group (LB, LC, and field) and for the total sample.

Groups	Diversity				
	n	N_A_	H_e_	H_o_	F_IS_
LB	4	2.00	0.314	0.365	−0.16
LC	3	1.67	0.272	0.259	0.05
Field	81	3.00	0.355	0.443	−0.25
Total/Mean	88	2.22	0.314	0.356	

n = number of samples; N_A_ = average number of alleles per locus; H_e_ = expected heterozygosity; H_o_ = observed heterozygosity; F_IS_ = inbreeding coefficient).

**Table 3:** Pairwise F_ST_ values among the three groups. Values in italic are significant according to a permutation test.

	LC	Field
LB	0.01	*0.159*
LC	-	*0.357*

LB = *L. barbarum* (different provenances), LC = *L. chinense* (different provenances), Field = accessions from the orchard.

**Table 4:** Bayesian analysis of the genetic structure of populations based on the three identified clusters.

	n.	Clusters		
		1	2	3
LB	4	0.008	0.254	0.739
LC	3	0.036	0.021	0.943
Field	81	0.458	0.534	0.008

**Table 5:** Information on the nine SSR microsatellite markers used, namely primer sequences (5′-3′ Fw and Rv), repeat motifs, melting temperature (T*_m_*, °C), size range (bp), number of alleles (A), indices of minor allele frequencies (MAF), expected heterozygosity (He), observed heterozygosity (Ho), and polymorphism index content (PIC).

Marker	Primer Sequence (5′-3′)		Repeat Motif	T*_m_* (°C)	Size Range (bp)	A	MAF	He	Ho	PIC
	**Forward**	**Reverse**								
GB-LCM-021	ATCAAGGCGCTATTTCCC	GGCCGGGATCTGTTAGAC	(AT)_4_	58	237–321	3	0.83	0.17	0.29	0.27
GB-LCM-025	AAGACAGCACGCCAAAAA	AGCCACCCCCAACTAAAA	(GAG)_4_	58	258–267	3	0.58	0.83	0.54	0.46
GB-LCM-075	TCTCCTTCGGACCCATTT	TTGGCATAAGGTGCTCGT	(CA)_15_	58	136–228	5	0.40	0.80	0.72	0.68
GB-LCM-087	CTCCTGAATACCCTGGGC	AGAAGAAGCAGCAGCACG	(GCW)_34_	58	117–240	5	0.58	0.50	0.61	0.58
GB-LCM-104	TTTGGAATGAAACGACGG	ACACCCCCGAGACTTAGC	(GTT)_2_, (GTT)_2_	58	289–346	3	0.58	0.50	0.57	0.50
GB-LCM-119	AATGTACATCGCCCCCA	GATTCGGAGCCTGCTTTT	(CA)_4_, (CA)_4_	58	276–286	3	0.50	1.00	0.57	0.48
GB-LCM-166	CTGAGAGCTGATGTGGC	AGGAGGAGAAGGGGGAAG	(TTC)_3_	58	213–225	3	0.50	1.00	0.58	0.49
GB-LCM-167	CTTGAAGATGGAGGAAAGCA	CCCAAAATTAAAGGGGCA	(GA)_7_, (GA)_18_	58	193–227	6	0.40	0.60	0.76	0.73
GB-LCM-199	CCATTTGCACCACAAAGG	TAAGGGCCCTCTTCAACG	(CA)_9_, (CA)_2_	58	293–317	3	0.67	0.67	0.49	0.42

## Data Availability

Data will be made available on request.

## Supplementary Materials

The following supporting information can be downloaded at: https://www.mdpi.com/article/10.3390/plants14081182/s1. Figure S1: Neighbor-Joining phylogenetic tree constructed using MEGA7. The tree procedure was solely for the purpose of facilitating visualization of the results obtained from the fingerprinting and understanding if each accession can be easily identified in comparison to the others. Table S1: List of the 88 *L. barbarum* and *L. chinense* accessions used in this study.

## References

[1] Hasan N., Choudhary S., Naaz N., Sharma N., Laskar R.A. (2021). Recent Advancements in Molecular Marker-Assisted Selection and Applications in Plant Breeding Programmes. J. Genet. Eng. Biotechnol..

[2] Amom T., Nongdam P. (2017). The Use of Molecular Marker Methods in Plants: A Review. Int. J. Curr. Res. Rev..

[3] Sharma R., Joshi A., Maloo S., Rajaman G. (2012). Assessment of Genetic Finger Printing Using Molecular Marker in Plants: A Review. Sci. Res. Impact.

[4] Idrees M., Irshad M. (2014). Molecular Markers in Plants for Analysis of Genetic Diversity: A Review. Eur. Acad. Res..

[5] Powell W., Machray G.C., Provan J. (1996). Polymorphism Revealed by Simple Sequence Repeats. Trends Plant Sci..

[6] Choudhary A., Kumar A., Kaur H., Balamurugan A., Padhy A.K., Mehta S. (2021). Plant Performance and Defensive Role of β-Amino Butyric Acid Under Environmental Stress. Plant Performance Under Environmental Stress.

[7] Kong Q., Zhang G., Chen W., Zhang Z., Zou X. (2012). Identification and Development of Polymorphic EST-SSR Markers by Sequence Alignment in Pepper, *Capsicum annuum* (Solanaceae). Am. J. Bot..

[8] Ismail N.A., Rafii M., Mahmud T., Hanafi M., Miah G. (2019). Genetic Diversity of Torch Ginger (*Etlingera elatior*) Germplasm Revealed by ISSR and SSR Markers. BioMed Res. Int..

[9] Wang Y., Rashid M.A.R., Li X., Yao C., Lu L., Bai J., Li Y., Xu N., Yang Q., Zhang L. (2019). Collection and Evaluation of Genetic Diversity and Population Structure of Potato Landraces and Varieties in China. Front. Plant Sci..

[10] Banks S.C., Cary G.J., Smith A.L., Davies I.D., Driscoll D.A., Gill A.M., Lindenmayer D.B., Peakall R. (2013). How Does Ecological Disturbance Influence Genetic Diversity?. Trends Ecol. Evol..

[11] Ramanatha Rao V., Hodgkin T. (2002). Genetic Diversity and Conservation and Utilization of Plant Genetic Resources. Plant Cell Tissue Organ Cult..

[12] Lee S.-R., Choi J.-E., Lee B.-Y., Yu J.-N., Lim C.E. (2018). Genetic Diversity and Structure of an Endangered Medicinal Herb: Implications for Conservation. AoB Plants.

[13] Zhang Y.-B., Shaw P.-C., Sze C.-W., Wang Z.-T., Tong Y. (2007). Molecular Authentication of Chinese Herbal Materials. J. Food Drug Anal..

[14] Amagase H., Farnsworth N.R. (2011). A Review of Botanical Characteristics, Phytochemistry, Clinical Relevance in Efficacy and Safety of *Lycium barbarum* Fruit (Goji). Food Res. Int..

[15] Potterat O. (2010). Goji (*Lycium barbarum* and *L. chinense*): Phytochemistry, Pharmacology and Safety in the Perspective of Traditional Uses and Recent Popularity. Planta Medica.

[16] Zhang K.Y., Leung H., Yeung H., Wong R.N. (2001). Differentiation of *Lycium barbarum* from Its Related Lycium Species Using Random Amplified Polymorphic DNA. Planta Medica.

[17] Sze S.C., Song J., Wong R.N., Feng Y., Ng T., Tong Y., Zhang K.Y. (2008). Application of SCAR (Sequence Characterized Amplified Region) Analysis to Authenticate *Lycium barbarum* (Wolfberry) and Its Adulterants. Biotechnol. Appl. Biochem..

[18] Kwon S.-J., Lee G.-A., Lee S.-Y., Park Y.-J., Gwag J.-G., Kim T.-S., Ma K.-H. (2009). Isolation and Characterization of 21 Microsatellite Loci in Lycium Chinense and Cross-Amplification in *Lycium barbarum*. Conserv. Genet..

[19] Chung J.-W., Lee G.-A., Lee S.-S., Bang K.-H., Park C.-B., Park Y.-J. (2009). Cultivar Discrimination of Korean and Chinese Boxthorn (*Lycium chinense* Mill. and *Lycium barbarum* L.) Using SSR Markers. Korean J. Med. Crop Sci..

[20] Zhao W.-G., Chung J.-W., Cho Y.-I., Rha W.-H., Lee G.-A., Ma K.-H., Han S.-H., Bang K.-H., Park C.-B., Kim S.-M. (2010). Molecular Genetic Diversity and Population Structure in Lycium Accessions Using SSR Markers. Comptes Rendus Biol..

[21] Gong H., Rehman F., Yang T., Li Z., Zeng S., Pan L., Li Y., Wang Y. (2019). Construction of the First High-Density Genetic Map and QTL Mapping for Photosynthetic Traits in *Lycium barbarum* L. Mol. Breed..

[22] Gao X., Li J., Song J., Guo Q. (2023). The SSR Genetic Diversity of Wild Red Fruit Lycium (*Lycium barbarum*) in Northwest China. Forests.

[23] Chen C., Xu M., Wang C., Qiao G., Wang W., Tan Z., Wu T., Zhang Z. (2017). Characterization of the *Lycium barbarum* Fruit Transcriptome and Development of EST-SSR Markers. PLoS ONE.

[24] Bertoldi D., Cossignani L., Blasi F., Perini M., Barbero A., Pianezze S., Montesano D. (2019). Characterisation and Geographical Traceability of Italian Goji Berries. Food Chem..

[25] Xin T., Yao H., Gao H., Zhou X., Ma X., Xu C., Chen J., Han J., Pang X., Xu R. (2013). Super Food *Lycium barbarum* (Solanaceae) Traceability via an Internal Transcribed Spacer 2 Barcode. Food Res. Int..

[26] Yao R., Heinrich M., Weckerle C.S. (2018). The Genus Lycium as Food and Medicine: A Botanical, Ethnobotanical and Historical Review. J. Ethnopharmacol..

[27] Kalia R.K., Rai M.K., Kalia S., Singh R., Dhawan A. (2011). Microsatellite Markers: An Overview of the Recent Progress in Plants. Euphytica.

[28] Wright S. (1984). Variability within and among Natural Populations. Evolution and the Genetics of Populations.

[29] Fukuda T., Yokoyama J., Ohashi H. (2001). Phylogeny and Biogeography of the Genus Lycium (Solanaceae): Inferences from Chloroplast DNA Sequences. Mol. Phylogenetics Evol..

[30] Zhang D., Xia T., Dang S., Fan G., Wang Z. (2018). Investigation of Chinese Wolfberry (*Lycium* Spp.) Germplasm by Restriction Site-Associated DNA Sequencing (RAD-Seq). Biochem. Genet..

[31] Poggioni L., Romi M., Guarnieri M., Cai G., Cantini C. (2022). Nutraceutical Profile of Goji (*Lycium barbarum* L.) Berries in Relation to Environmental Conditions and Harvesting Period. Food Biosci..

[32] Doyle J., Doyle J.L. (1987). Genomic Plant DNA Preparation from Fresh Tissue-CTAB Method. Phytochem. Bull..

[33] Schuelke M. (2000). An Economic Method for the Fluorescent Labeling of PCR Fragments. Nat. Biotechnol..

[34] Weir B., Cockerham C.C. (1984). Estimating F-Statistics for the Analysis of Population Structure. Evolution. Estim. F-Stat. Anal. Popul. Struct. Evol..

[35] Nei M. (1978). Estimation of Average Heterozygosity and Genetic Distance from a Small Number of Individuals. Genetics.

[36] Belkhir K. (1999). GENETIX, Logiciel Sous WindowsTM Pour La Génétique Des Populations. https://kimura.univ-montp2.fr/genetix/.

[37] Pritchard J.K., Stephens M., Donnelly P. (2000). Inference of Population Structure Using Multilocus Genotype Data. Genetics.

[38] Evanno G., Regnaut S., Goudet J. (2005). Detecting the Number of Clusters of Individuals Using the Software STRUCTURE: A Simulation Study. Mol. Ecol..

[39] Earl D.A., VonHoldt B.M. (2012). STRUCTURE HARVESTER: A Website and Program for Visualizing STRUCTURE Output and Implementing the Evanno Method. Conserv. Genet. Resour..

[40] Pagnotta M.A. (2018). Comparison among Methods and Statistical Software Packages to Analyze Germplasm Genetic Diversity by Means of Codominant Markers. J.

[41] Kumar S., Stecher G., Tamura K. (2016). MEGA7: Molecular Evolutionary Genetics Analysis Version 7.0 for Bigger Datasets. Mol. Biol. Evol..

