# Supplementary material for: Molecular Analysis by Microsatellite Markers of Goji Plants (Lycium barbarum L.) Grown in Central Italy Reveal Genetic Distinction from Both L. barbarum and L. chinense Species
Source: Plants (Basel). 2025 Apr 10;14(8):1182. doi: 10.3390/plants14081182 (PMC12030364; doi:10.3390/plants14081182)
Supplement: Supplementary file 1 [file plants-14-01182-s001.zip › Supplementary materials_Figure S1.pdf]

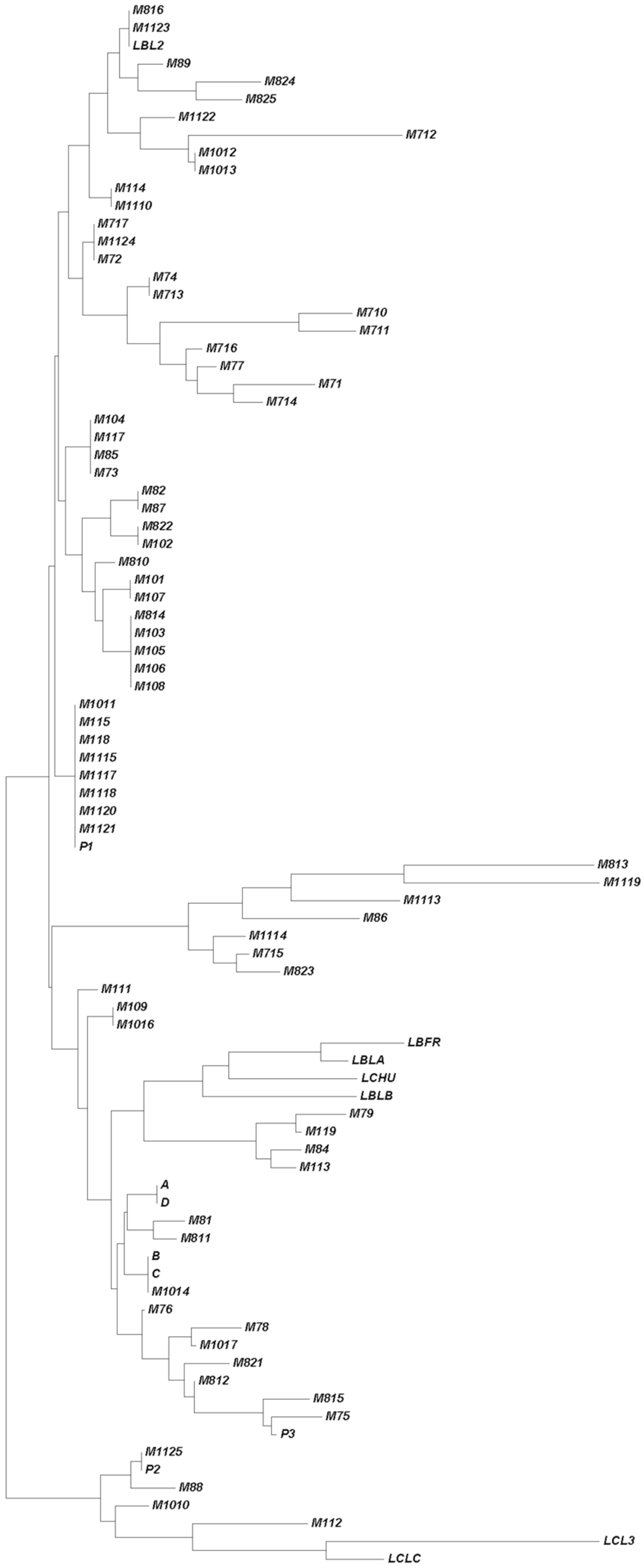

Figure S1. Neighbor-Joining phylogenetic tree constructed using MEGA7. The tree procedure was solely for the purpose of facilitating visualization of the results obtained from the fingerprinting and understanding if each accession can be easily identified in comparison to the others.
