# Supplementary material for: Molecular Analysis by Microsatellite Markers of Goji Plants (Lycium barbarum L.) Grown in Central Italy Reveal Genetic Distinction from Both L. barbarum and L. chinense Species
Source: Plants (Basel). 2025 Apr 10;14(8):1182. doi: 10.3390/plants14081182 (PMC12030364; doi:10.3390/plants14081182)
Supplement: Supplementary file 1 [file plants-14-01182-s001.zip › Supplementary materials_Table S1.pdf]

Table S1. List of the 88 *L. barbarum* and *L. chinense* accessions used in this study. The following information (column) is shown for each accession: sample ID (“Sample”); species of belonging, real or supposed (“Species”); site of sampling (“Collection site”); origin of the seeds or seedling (“Origin”).

Sample	Species	Collection site	Origin
A	*L. barbarum*	Bragaglia Farm	seedling from Vita Verde nursery
B	*L. barbarum*	Bragaglia Farm	seedling from Vita Verde nursery
C	*L. barbarum*	Bragaglia Farm	seedling from Vita Verde nursery
D	*L. barbarum*	Bragaglia Farm	seedling from Vita Verde nursery
P1	*L. barbarum*	Bragaglia Farm	seedling from Vita Verde nursery
P2	*L. barbarum*	Bragaglia Farm	seedling from Vita Verde nursery
P3	*L. barbarum*	Bragaglia Farm	seedling from Vita Verde nursery
M7-1	*L. barbarum*	Bragaglia Farm	seedling from Vita Verde nursery
M7-2	*L. barbarum*	Bragaglia Farm	seedling from Vita Verde nursery
M7-3	*L. barbarum*	Bragaglia Farm	seedling from Vita Verde nursery
M7-4	*L. barbarum*	Bragaglia Farm	seedling from Vita Verde nursery
M7-5	*L. barbarum*	Bragaglia Farm	seedling from Vita Verde nursery
M7-6	*L. barbarum*	Bragaglia Farm	seedling from Vita Verde nursery
M7-7	*L. barbarum*	Bragaglia Farm	seedling from Vita Verde nursery
M7-8	*L. barbarum*	Bragaglia Farm	seedling from Vita Verde nursery
M7-9	*L. barbarum*	Bragaglia Farm	seedling from Vita Verde nursery
M7-10	*L. barbarum*	Bragaglia Farm	seedling from Vita Verde nursery
M7-11	*L. barbarum*	Bragaglia Farm	seedling from Vita Verde nursery
M7-12	*L. barbarum*	Bragaglia Farm	seedling from Vita Verde nursery
M7-13	*L. barbarum*	Bragaglia Farm	seedling from Vita Verde nursery
M7-14	*L. barbarum*	Bragaglia Farm	seedling from Vita Verde nursery
M7-15	*L. barbarum*	Bragaglia Farm	seedling from Vita Verde nursery
M7-16	*L. barbarum*	Bragaglia Farm	seedling from Vita Verde nursery
M7-17	*L. barbarum*	Bragaglia Farm	seedling from Vita Verde nursery
M8-1	*L. barbarum*	Bragaglia Farm	seedling from Vita Verde nursery
M8-2	*L. barbarum*	Bragaglia Farm	seedling from Vita Verde nursery
M8-4	*L. barbarum*	Bragaglia Farm	seedling from Vita Verde nursery
M8-5	*L. barbarum*	Bragaglia Farm	seedling from Vita Verde nursery
M8-6	*L. barbarum*	Bragaglia Farm	seedling from Vita Verde nursery
M8-7	*L. barbarum*	Bragaglia Farm	seedling from Vita Verde nursery
M8-8	*L. barbarum*	Bragaglia Farm	seedling from Vita Verde nursery
M8-9	*L. barbarum*	Bragaglia Farm	seedling from Vita Verde nursery
M8-10	*L. barbarum*	Bragaglia Farm	seedling from Vita Verde nursery
M8-11	*L. barbarum*	Bragaglia Farm	seedling from Vita Verde nursery
M8-12	*L. barbarum*	Bragaglia Farm	seedling from Vita Verde nursery
M8-13	*L. barbarum*	Bragaglia Farm	seedling from Vita Verde nursery
M8-14	*L. barbarum*	Bragaglia Farm	seedling from Vita Verde nursery
M8-15	*L. barbarum*	Bragaglia Farm	seedling from Vita Verde nursery
M8-16	*L. barbarum*	Bragaglia Farm	seedling from Vita Verde nursery
M8-21	*L. barbarum*	Bragaglia Farm	seedling from Vita Verde nursery
M8-22	*L. barbarum*	Bragaglia Farm	seedling from Vita Verde nursery
M8-23	*L. barbarum*	Bragaglia Farm	seedling from Vita Verde nursery
M8-24	*L. barbarum*	Bragaglia Farm	seedling from Vita Verde nursery
M8-25	*L. barbarum*	Bragaglia Farm	seedling from Vita Verde nursery
M10-1	*L. barbarum*	Bragaglia Farm	seedling from Vita Verde nursery
M10-2	*L. barbarum*	Bragaglia Farm	seedling from Vita Verde nursery

M10-3	*L. barbarum*	Bragaglia Farm	seedling from Vita Verde nursery
M10-4	*L. barbarum*	Bragaglia Farm	seedling from Vita Verde nursery
M10-5	*L. barbarum*	Bragaglia Farm	seedling from Vita Verde nursery
M10-6	*L. barbarum*	Bragaglia Farm	seedling from Vita Verde nursery
M10-7	*L. barbarum*	Bragaglia Farm	seedling from Vita Verde nursery
M10-8	*L. barbarum*	Bragaglia Farm	seedling from Vita Verde nursery
M10-9	*L. barbarum*	Bragaglia Farm	seedling from Vita Verde nursery
M10-10	*L. barbarum*	Bragaglia Farm	seedling from Vita Verde nursery
M10-11	*L. barbarum*	Bragaglia Farm	seedling from Vita Verde nursery
M10-12	*L. barbarum*	Bragaglia Farm	seedling from Vita Verde nursery
M10-13	*L. barbarum*	Bragaglia Farm	seedling from Vita Verde nursery
M10-14	*L. barbarum*	Bragaglia Farm	seedling from Vita Verde nursery
M10-16	*L. barbarum*	Bragaglia Farm	seedling from Vita Verde nursery
M10-17	*L. barbarum*	Bragaglia Farm	seedling from Vita Verde nursery
M11-1	*L. barbarum*	Bragaglia Farm	seedling from Vita Verde nursery
M11-2	*L. barbarum*	Bragaglia Farm	seedling from Vita Verde nursery
M11-3	*L. barbarum*	Bragaglia Farm	seedling from Vita Verde nursery
M11-4	*L. barbarum*	Bragaglia Farm	seedling from Vita Verde nursery
M11-5	*L. barbarum*	Bragaglia Farm	seedling from Vita Verde nursery
M11-7	*L. barbarum*	Bragaglia Farm	seedling from Vita Verde nursery
M11-8	*L. barbarum*	Bragaglia Farm	seedling from Vita Verde nursery
M11-9	*L. barbarum*	Bragaglia Farm	seedling from Vita Verde nursery
M11-10	*L. barbarum*	Bragaglia Farm	seedling from Vita Verde nursery
M11-13	*L. barbarum*	Bragaglia Farm	seedling from Vita Verde nursery
M11-14	*L. barbarum*	Bragaglia Farm	seedling from Vita Verde nursery
M11-15	*L. barbarum*	Bragaglia Farm	seedling from Vita Verde nursery
M11-17	*L. barbarum*	Bragaglia Farm	seedling from Vita Verde nursery
M11-18	*L. barbarum*	Bragaglia Farm	seedling from Vita Verde nursery
M11-19	*L. barbarum*	Bragaglia Farm	seedling from Vita Verde nursery
M11-20	*L. barbarum*	Bragaglia Farm	seedling from Vita Verde nursery
M11-21	*L. barbarum*	Bragaglia Farm	seedling from Vita Verde nursery
M11-22	*L. barbarum*	Bragaglia Farm	seedling from Vita Verde nursery
M11-23	*L. barbarum*	Bragaglia Farm	seedling from Vita Verde nursery
M11-24	*L. barbarum*	Bragaglia Farm	seedling from Vita Verde nursery
M11-25	*L. barbarum*	Bragaglia Farm	seedling from Vita Verde nursery
LCHU	*L. chinense*	CNR S. Paolina Farm	Hungary 1464 (HUN)
LCL3	*L. chinense*	CNR S. Paolina Farm	Ljubljana Botanical Garden (SLO)
LCLC	*L. chinense*	CNR S. Paolina Farm	A.Di.P.A. Lucca (ITA)
LBLB	*L. barbarum*	CNR S. Paolina Farm	A.Di.P.A. Lucca (ITA)
LBFR	*L. barbarum*	CNR S. Paolina Farm	Tours Botanical Garden (FRA)
LBL2	*L. barbarum*	CNR S. Paolina Farm	Mainz Botanical Garden (GER)
LBLA	*L. barbarum*	CNR S. Paolina Farm	Salapilis Botanical Garden (LAT)
